# The Effect of Calcination Temperature on the Structure and Performance of Nanocrystalline Mayenite Powders

**DOI:** 10.3390/ma12213476

**Published:** 2019-10-24

**Authors:** Katarzyna Berent, Sebastian Komarek, Radosław Lach, Waldemar Pyda

**Affiliations:** 1 Academic Centre for Materials and Nanotechnology, AGH University of Science and Technology, 30 Mickiewicza Av., 30-059 Kraków, Poland; 2 Faculty of Materials Science and Ceramics, AGH University of Science and Technology, 30 Mickiewicza Av., 30-059 Kraków, Poland; seko@agh.edu.pl (S.K.); rlach@agh.edu.pl (R.L.); pyda@agh.edu.pl (W.P.)

**Keywords:** calcination temperature, calcium aluminate, mayenite, thermal behavior, nanocrystalline powder, microstructure

## Abstract

The effect of calcination temperature on the structural properties and phase formation of synthesized CaO-Al_2_O_3_ nanopowder was investigated and discussed. The calcination products were identified by differential thermal analysis (DTA) and the crystalline phase formation was analyzed by X-ray diffraction (XRD). The obtained results showed that the crystallization started at 460 °C. Finally, the microstructures of the nanoparticles were observed by scanning (SEM) and transmission electron (TEM) microscopes. The investigation showed that an increase in the calcination temperature led to the appreciable increase in the crystallite size and the crystallinity of the final product. The obtained data confirmed that the prepared materials were mayenite with different surface area in the range of 71.18 m^2^/g to 10.34 m^2^/g after annealing in the temperature range of 470 °C to 960 °C.

## 1. Introduction

The calcium aluminates, CaO-Al_2_O_3_ (C-A) system is a promising group of materials due to superior refractory properties. Especially, mayenite (12CaO·7Al_2_O_3_ labelled as C12A7) is an auspicious functional material for usage in various engineering applications, such as catalysis [1,2,3,4], in particular, used in the synthesis of ammonia [5,6], batteries [7], white light-emitting diodes (W-LEDs) [8], electronic [9] and optoelectronic devices [10], which results from the discovery of oxygen mobility [11] and ionic conductivity [12,13,14]. The unique properties of C12A7 come from its special crystal structure. The unit cell contains two formula units (Z = 2) and is convenient to define as [Ca_24_Al_28_O_64_]^4+^ + 2O^2−^ [14]. It is composed of a Ca-Al-O framework forming interconnected 12 cages with a positive electric charge, the extra-framework highly mobile two oxygen ions occupy statistically two of these cages and compensate the charge of the framework [15,16]. On the one hand, the substitution for the free oxygen ions O^2−^ by F^−^, Cl^−^ and OH^−^ induces C12A7 into an electride in which electrons behave as anions [14].

The engineering applications of C-A powders depend considerably on their crystallite size and composition which are determined by the rate and temperature of calcination during synthesis [17]. Various preparation techniques, such as solid state routes [18], sol-gel [19,20,21], hydrothermal followed by calcination [17,22], the Pechini process [23], oxalate-precursor co-precipitation [24], the glycine/nitrate procedure-GNP [23,25], the spray pyrolysis method [26] and synthesis based on polymethyl methacrylate (PMMA) as a soft templating agent [27] are employed to prepare calcium aluminate powders composed of nanoparticles (NPs).

There is a growing interest in the synthesis of NPs in recent years, that possess unique physical and chemical properties due to their extremely high surface area and associated nanoscale size, which opens new possibilities for their applications. At these reduced dimensions, NPs provide a tremendous driving force for sintering, shorter distances for mass transportation and hence the surface energy of the nanoparticles substantially affects the interior bulk properties of the resultant materials [28]. In the case of NPs, the sintering can take place at lower temperatures, over shorter time scales, when compared to larger particles. Since the physical properties of ceramics are very sensitive to material density and microstructure, understanding the control of the above properties of the ceramics with respect to the ceramic processing parameters affected by sintering, such as a powder morphology, particles size, density and the sintering temperature is essential. Depending on the future application of C-A, the final morphology of the powder can be controlled. The sintering rate determines the growth rate of nanoparticles and is crucial for the design of their synthesis with controlled size, structure, composition. It is therefore, the objective of this study to investigate the effects of modification of calcination conditions on calcium aluminate phase transition, powder purity, mean size and morphology.

In the present work, we have employed a novel variant of the conventional method to synthesize nanocrystalline powders which combine the use of nanometric boehmite particles and submicron calcium hydroxide. The main advantage of this synthesis over the conventional ceramic process of the solid state reaction process is the decreased temperature of reaction and nanometric size of the powder. The microstructure of the synthesized powders can be modified by changing reaction conditions to achieve the desired final product. Several analytical techniques such as thermogravimetric (TG/DTA), X-ray diffraction (XRD), transmission and scanning electron microscopy (TEM, SEM) were used to understand the changes in the synthesized material during the thermal calcination process.

## 2. Materials and Methods

The new combinational method of production of CaO-Al_2_O_3_ nanopowders under study comprised several steps. In the first one, a boehmite gel (γ-AlO(OH)) was obtained using nanocrystalline (20 nm) dawsonite (NH_4_AlCO_3_(OH)_2_), synthesized from aqueous saturated solutions of aluminium nitrate enneahydrate (Al(NO_3_)_3_·9H_2_O) (p.a.) and ammonium carbonate ((NH_4_)_2_CO_3_) (p.a.), which was subjected to a hydrothermal treatment in a stainless-steel autoclave for 2 h at 130 °C at 100 kPa pressure. Then, the formed nanocrystalline γ-AlO(OH) (crystallite size 3.7 nm), being in the form of a gel-like aqueous colloidal suspension, was mixed with Ca(OH)_2_ (p.a.) particles in a molar ratio of 14:12. The reactants were homogenized using a mixer mill at RT, dried in a laboratory dryer at 100 °C for constant weight and again ground for 15 min in a corundum mortar. Finally, the obtained powder was calcined in a conventional electric furnace for 30 min at different temperatures of 170 °C, 470 °C, 760 °C, 960 °C and 1250 °C under air atmosphere.

The thermogravimetric (TG/DTA) curves were recorded on a simultaneous thermal analyzer (STA 449 F3, NETZSCH, Selb, Germany). The instrument was operated from ambient to 1400 °C at a heating rate of 10 °C/min in atmospheric air.

The phase identification was performed by X-ray diffraction using an X-ray diffractometer (Empyrean, Malvern Panalytical, Malvern, UK) with Cu Kα radiation (*λ* = 1.5406 Å). Data were collected in the 2θ range of 10–89.90° with a resolution of 0.008°. The crystallite size of the calcined powders was determined by the X-ray line broadening technique performed on the most intense diffraction peak from the (420) plane of mayenite using the Scherrer equation [29]:

(1) $$D=\;\frac{0.9\lambda }{\sqrt{\beta_{sample}^{2}-\beta_{ref\;}^{2}}\;cos\theta }$$

where D is the crystallite size (nm), λ is the wavelength of CuKα radiation, which equals to 0.154 nm, β_sample_ is the FWHM of a diffraction peak and β_ref_ corresponds to the instrumental FWHM and θ is the Bragg angle.

The morphological structure was studied using a scanning electron microscope (Versa 3D, FEI Company, Hillsboro, OR, USA) whereas the exact dimensions of the nanopowders were investigated using transmission electron microscopy (Tecnai 200, FEI Company, Hillsboro, OR, USA). The samples for SEM and TEM were prepared by dispersing the calcined powder particles in alcohol and then dropped onto a carbon film supported on a copper grid. The TEM image analysis was done by measuring two perpendicular diameters from the particle with ImageJ software and then the diameter was calculated as the average dimension along the two axes.

The Brunauer–Emmer–Teller (BET) surface area was determined by means of the adsorption/desorption isotherms of liquid nitrogen at −196 °C using an automatic analyzer (ASAP 2000, Micromeritics Inc. Corp., Norcross, GA, USA).

## 3. Results and Discussion

### 3.1. Thermal Behaviour of the Precursor Mixture

**Figure 1:**
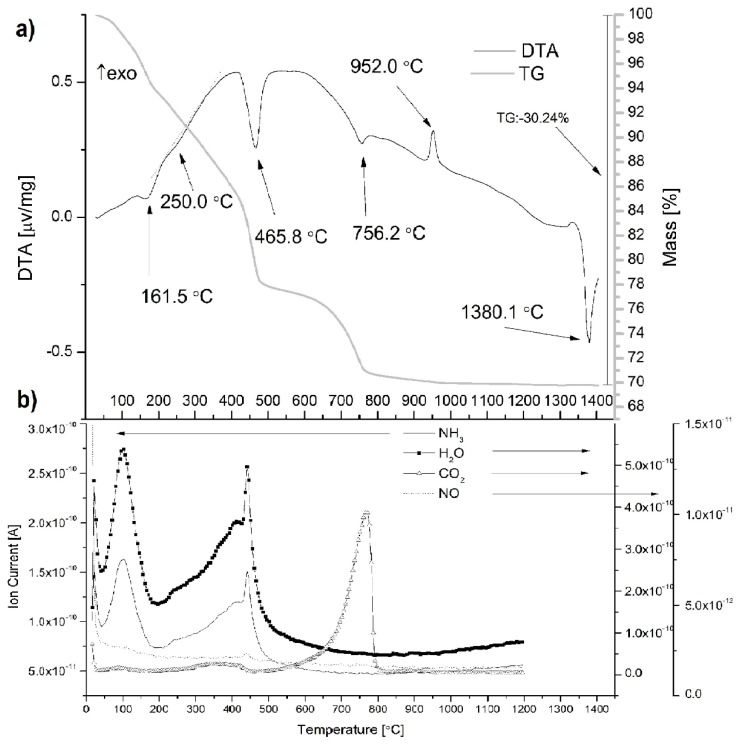
TG/DTA curves (**a**), and selected curves of mass-spectroscopic ion currents (**b**) for gases evolved during heating from the as-synthesized powder.

The TG/DTA technique was very useful in the characterization of the thermal behavior of the mayenite precursor mixture. Figure 1a shows the thermal evolution of the temperature range of RT to 1400 °C. Mass spectrometry (MS) results that present ion currents of NH_3_, H_2_O, CO_2_ and NO in gases released from samples during the TG/DTA experiment are additionally shown in Figure 1b.

**Figure 2:**
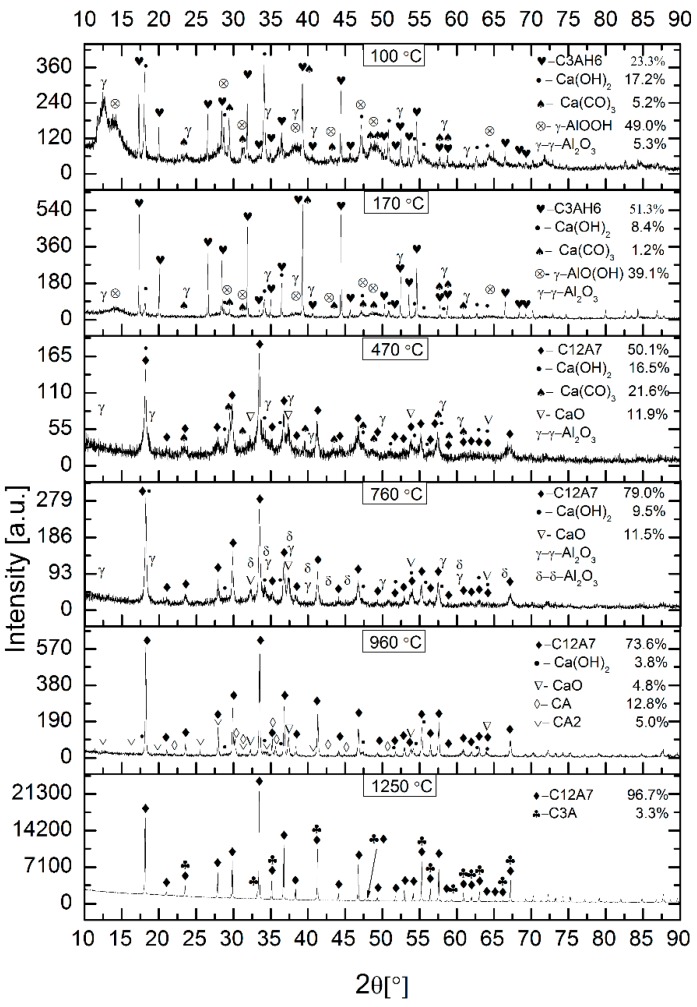
XRD patterns of powders calcined at different temperatures; phase names are indicated together with their weight contents.

A correct interpretation of the results of TG/DTA and released gases requires knowledge of the phase composition of the tested mixture of mayenite precursors, which is given in Figure 2. The main components of this mixture, which was dried to constant weight at 100 °C, were nano-boehmite and calcium hydroxide, but the hydrogranet phase (Ca_3_Al_2_(OH)_12_, C3AH6) in the amount of 23.3 wt %, which is already formed at 45 °C according to [30], was also found. Calcium carbonate was introduced into the mixture together with calcium hydroxide in which it was present in an amount of 3.99%, as revealed by TG/DTA analysis of this reagent. The probable presence of γ-Al_2_O_3_ would be associated with the dehydration of some nano-boehmite crystallites.

Essentially six endothermic peaks are observed: extremely weak at 100 °C, then pronounced at 161.5 °C, blurred at ~250 °C, intense at 465.8 °C and weaker at 756.2 °C; these effects are accompanied by correlated weight losses, as shown by the TG curve. The last endothermic peak occurs at 1380.1 °C, and the sample does not change its mass. The exothermic peak is observed only at 952 °C.

Several components, such as the physically adsorbed species like water and organic molecules are removed from the system at the lower temperature range [31,32]. The maxima on the H_2_O, NH_3_ and CO_2_ ion current curves at 100 °C (Figure 1b), which correspond to the extremely poorly marked endothermic peak on the DTA curve (Figure 1a), should be attributed to the desorption of these molecules from the surface of the particles of the phases that formed the mixture of mayenite precursors.

The second endothermic peak appears at 161.5 °C, which is attributed to a small mass loss. The initial decrease in mass occurs after the beginning of the heating stage it is related to the beginning of the reaction of forming additional portions of C3AH6 from unreacted nano-boehmite and calcium hydroxide particles according to the following equation [6]:

(2) $$2\;\gamma \mathrm{AlO}\left(\mathrm{OH}\right)+3\mathrm{Ca}{\left(\mathrm{OH}\right)}_{2}+\;2H_{2}O\to {\;\mathrm{Ca}}_{3}{\mathrm{Al}}_{2}{\left(\mathrm{OH}\right)}_{12}$$

The highest rate of hydrogarnet formation falls at 161.5 °C. Since this reaction consumes water molecules in the range from ~45 °C to ~200 °C, the ion current for water molecules is the combined effect of water desorption processes and hydrogarnet formation.

The third broad and weak endothermic peak appears at 250 °C, due to the dehydration of C3AH6, which leads to a transition into C12A7, which is the hydrated state described as Ca_12_Al_14_O_33_∙H_2_O (C12A7H). Simultaneously Ca(OH)_2_ is formed and is further converted to CaO (and CaCO_3_ in air) above ~350 °C [6]. The reaction of forming hydrated C12A7 is presented in the following equation:

(3) $$7{\mathrm{Ca}}_{3}{\mathrm{Al}}_{2}{\left(\mathrm{OH}\right)}_{12}\to {\mathrm{Ca}}_{12}{\mathrm{Al}}_{14}O_{33}\cdot H_{2}O+9\mathrm{Ca}{\left(\mathrm{OH}\right)}_{2}+32H_{2}O↑$$

The proposed reaction is in line with the increase in the ionic current of the released water molecules, which is observed in the hydrogarnet dehydration temperature range. In this range, NH_3_ and CO_2_ molecules are also released, proving their binding to the structure of formed mayenite crystallites.

The forth endothermic peak emerging at about 465.8 °C is due to the dehydration of Ca(OH)_2_ to yield CaO according to the reaction [33]:

(4) $$2\mathrm{Ca}{\left(\mathrm{OH}\right)}_{2}\to 2\mathrm{CaO}+2H_{2}O↑$$

This endothermic event is accompanied by weight loss, as well as the release of H_2_O, NH_3_ and additionally NO molecules (Figure 1b). The temperature of this peak agrees well with a temperature of 468 °C determined in a separate TG/DTA measurement for the decomposition of calcium hydroxide. This study also revealed the presence of 3.99% calcium carbonate, which decomposed at 703 °C.

The fifth endothermic peak around 756.2 °C is assigned to the decomposition of CaCO_3_ according to the reaction:

(5) $${\mathrm{CaCO}}_{3}\;\to \;\mathrm{CaO}+{\mathrm{CO}}_{2}↑$$

This peak is accompanied by weight loss and CO_2_ release, as shown in Figure 2a,b, respectively. Primary CaCO_3_ contained in the initial calcium hydroxide and secondary CaCO_3_ derived from the decomposition of the hydrogarnet gave a contribution to the observed thermal effect.

It can be concluded that the formation of monocalcium aluminate (CaAl_2_O_4_, CA) is observed at 952.0 °C, as revealed by the XRD patterns [20].

(6) $$5{\mathrm{Al}}_{2}O_{3}+{\mathrm{Ca}}_{12}{\mathrm{Al}}_{14}O_{33}\;\to \;12{\mathrm{CaAl}}_{2}O_{4}$$

The sixth endothermic peak emerging at about 1380.1 °C is attributed to the melting of Ca_12_Al_14_O_33_, which indicates that the phase transformation occurred.

### 3.2. Phase Analysis of the Product

The component phases of the product were examined with XRD, and qualitative agreement with literature data was obtained. Figure 2 shows the XRD patterns of the as-prepared mixture and those calcined at 100 °C, 170 °C, 470 °C, 760 °C, 960 °C and 1250 °C, respectively.

For the sample annealed at 170 °C, there are peaks corresponding to the form of C3AH6, Ca(OH)_2_, Ca(CO)_3_, γ-AlO(OH). Aluminium phase with a structure similar to γ-Al_2_O_3_ is probably present at this temperature. However, the indexing is difficult due to the low intensity peaks. C3AH6 formation during heat treatment at 161.5 °C in accordance with reaction (1) is marked by the increase in the amount compared to the initial sample of the precursor mixture and the simultaneous increase in the content of boehmite and calcium hydroxide.

After calcination at 470 °C, complete disappearance of the hydrogarnet is observed along with the appearance of the mayenite phase, probably hydrated (C12A7H) together with CaO and some amounts of Ca(OH)_2_, Ca(CO)_3_, and γ-Al_2_O_3_. Lack of anhydrous hydrograrnet phase [17,32,34,35] in the sample heat treated at 170 °C and increased the content of calcium hydroxide, calcium carbonate and calcium oxide in the sample from 470 °C suggests the mechanism of mayenite formation. According to this mechanism (Equation (2)) the topotactic decomposition of hydrogarnet occurs at temperature exceeding ~220 °C, as indicated by TG/DTA measurements. The presence of significant amounts of calcium hydroxide indicates only its partial decomposition under used heat treatment conditions.

At higher temperatures, γ-Al_2_O_3_ decomposes into δ-Al_2_O_3_ [32], and the formation of CaO from the decomposition of calcium carbonate is observed. Thus, increasing the temperature of the heat treatment of the precursor mixture to 760 °C resulted in the complete decomposition of Ca(CO)_3_, a significant reduction in the calcium hydroxide content with a slight change in the CaO content, but also a significant increase in the C12A7 content by ~30 wt %. The phases γ-Al_2_O_3_ and δ-Al_2_O_3_ occurred in an amount impossible to measure reliably. It should be noted, therefore, that failure to include aluminium phases in the phase composition of the 470 °C and 760 °C samples means that the calculations of the component phase contents are only indicative and allow only a qualitative interpretation of these results. There is no doubt that the increase in C12A7’s content in the absence of a hydrogarnet as a precursor suggests a mechanism responsible for this fact other than topotactic degradation of the hydrogarnet. The results of X-ray studies indicate a direct reaction of oxide precursors of the C12A7 phase with the participation of solid state diffusion and the use of the process of nucleating new crystallites on the surfaces of already existing crystalline mayenite particles or growing atom by atom of these surfaces. Often the presence of CA may hasten the set time of the CA2 phase, which is why it appears in aluminous cements as a secondary phase [36].

Further, by increasing the calcination temperature to 960 °C, the XRD pattern indicates the formation of new aluminate phases: CA and monocalcium dialuminate (CaAl_4_O_7_, CA2) at the expense of a significant reduction in the content of calcium hydroxide and calcium oxide with virtually unchanged contribution of C12A7 (this result may be underestimated due to the fact that the unmeasured amount of aluminium oxide was not included in the calculations). This clear variation in the phase composition may have its source in the initial heterogeneity of the mixture of nano-bohemite and calcium hydroxide much coarser. This heterogeneity could even deepen during the formation of phase composition.

The highest heat treatment temperature used was 1250 °C had a very positive effect on the homogeneity of the phase composition. The content of C12A7 increased to 96.7 wt %, and as the second phase, tricalcium aluminate (3CaO∙Al_2_O_3_, C3A) is present in the amount of 3.3 wt %, which is formed in the reaction between C12A17 and CaO [37].

The width of the diffraction peaks of the mayenite phase (Figure 2) and data of Table 1 confirm the nanocrystalline nature of the prepared materials. The crystallite size of primary particles is measured by the XRD method and they are higher as the calcination temperature increases. The thermodynamically justified phenomenon of crystallite size growth with increasing calcination temperature is associated with the enhancement of the ratio of crystalline volume to the surface, which occurs as a result of enlargement of the particle size, reducing the Gibbs free energy of the system [38].

As shown in Table 1, the average crystallite size D_XRD_ of mayenite particles increases with the increase of the calcination temperature from 31.1 nm to 81.3 nm in the studied temperature range. By comprehensive consideration, the optimal calcination temperature for the precursor mixture used is 470 °C, due to the appropriate crystallinity fraction and the smallest crystallite size.

### 3.3. Microstructural Characterizations

**Figure 3:**
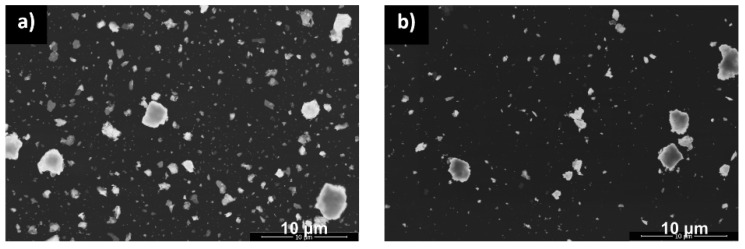

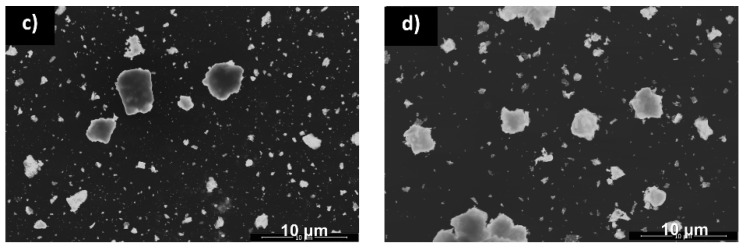
SEM micrographs of the powders calcined at different temperatures 170 °C (**a**), 470 °C (**b**), 760 °C (**c**) and 960 °C (**d**).

The morphologies of the four examined powders obtained at various temperatures are shown in the micrographs in Figure 3.

The formation of the aggregates from the primary particles is observed for all samples. A worm-like structure of primary particles can be noticed with large, irregularly shaped particles with 0.2–3.4 μm in diameter. Although the XRD analysis results indicate that the crystallite size increases with the increase of calcination temperature, this regulation cannot be directly recognized from the SEM images due to limitations of the method. TEM was used to improve the spatial resolution and the image quality of the primary crystallites.

**Figure 4:**
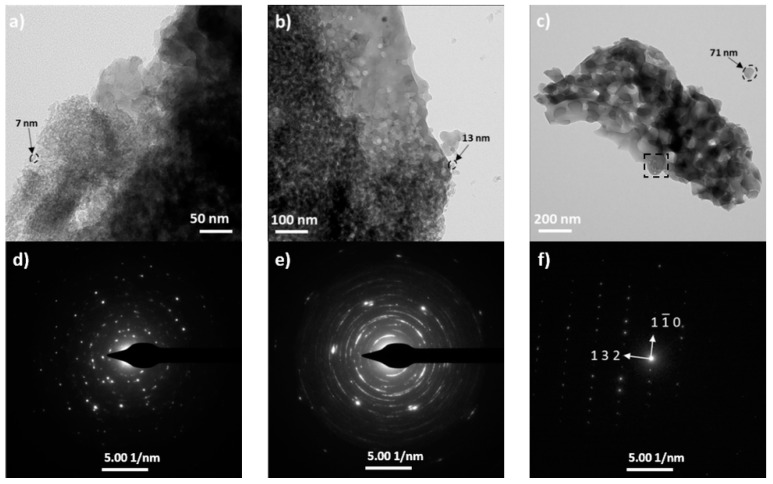
TEM micrographs and the corresponding SAED patterns of the powders calcined at 470 °C (**a**,**d**), 760 °C (**b**,**e**) and 960 °C (**c**,**f**).

The selected samples were subjected to TEM analysis for a better understanding of the effects of the calcination process on the morphology and phase formation of nanopowders. Figure 4 shows the bright field (BF) images and the corresponding selected area electron diffraction (SAED) patterns of the as-prepared powders calcined at 470 °C, 760 °C and 960 °C, respectively.

It can be observed that the primary particle morphology is nearly spherical and of uniform size with clear aggregation. The BF image of the sample calcined at 470 °C (Figure 4a) shows that it contains nanoparticles having sizes of ~5–13 nm whereas the powder calcined at 760 °C (Figure 4b) consists of particles of ~10–25 nm in diameter. The primary particles obtained in the lower temperatures show a narrow size distribution. Sinter necks are not observed for the samples sintered at lower temperatures. When raising the calcination temperature for a fixed isothermal time, the grain size of the CA grew significantly. The nanoparticles calcined at 960 °C consisted of large grains such as spheroidal shape particles that the surfaces of several neighboring particles were melted during the procedure due to the high temperature value. Under this condition, the primary crystallites are bonded together by solid bridges (typically formed during the calcination process). The BF micrograph (Figure 4c) reveals the presence of nonuniform aggregates and an internal porosity. Different types of aggregation are marked by a black square in the BF images. The SAED patterns (Figure 4d–f) showing diffuse rings and regular spots which confirms the crystallinity of the powders and is in good agreement with the XRD patterns. The primary particles obtained at lower temperatures turn out a continuous ring pattern due to the presence of a large number of single crystallites in the selected area. However, when the primary particle size increases at higher calcination temperature, the spots are far from each other. The crystal structure analysis using SAED fitted to the cubic crystal structure of mayenite with the *I-43d* space group and lattice parameters a = 11.982 nm (Figure 4c). It has a narrow particle size distribution ranging between 10.7 and 78.1 nm. The average TEM particle size of the nanocrystallites is shown in Table 1. The observed (sub)micrometric particles by SEM are porous agglomerates of nanometric primary crystallites as confirmed by the TEM studies. At an increasing calcination temperature, a continuous agglomeration of primary particles ensued and when the agglomeration is concluded, the rapid growth of isolated and connected particle was observed. Therefore, at the highest calcination temperature, the pores in the materials are trapped as a result of the sintering.

The relation between the BET surface area (S_BET_) and the calcination temperature of the powders is shown in Table 1. It can be noticed that the powder calcined at 470 °C has the highest BET surface area equal to 71.18 m^2^/g. The values gradually decrease with increasing calcination temperature and for heating the powder at 960 °C has 10.34 m^2^/g. The observed decrease in the SBET can be attributed to the increase in the crystallite size as confirmed by the XRD and TEM results. The obtained results are in accordance with the works of other authors. Li et al. report that the specific surface area of the mayenite prepared by the hydrothermal method and calcined at 400 °C for 4 h amounted to about 67.1 m^2^/g [22]. A novel route based on the use of polymethyl methacrylate (PMMA) described by Intiso et al. [27] allows to obtain the mayenite powder with the largest surface area of 47.1 m^2^/g for mayenite prepared using 10% w/w of PMMA. The crystallite size (D_XRD_) and the particle size (D_TEM_) data show some discrepancies, indicating that the primary crystallites are significantly agglomerated. The results show that the crystallite growth may be divided into three different ranges between 470 °C and 960 °C. In the temperature of 470 °C, where the nanoporous structure still remains intact, the crystallite growth was slow and the BET surface area remained the highest value. Then, at a temperature of 760 °C, an increase of the primary crystallite size and degree of agglomeration is observed. Finally, the melting of the particles and sintering occurred at a temperature of 960 °C. That is the reason why the crystallite size D_XDR_ shows larger values than that expected from the TEM analysis, which might be associated with a highly agglomerated structure observed in the TEM micrographs [39]. The differences in the primary particles sizes can be envisaged taking into account that the TEM images show the real powder particles size while XRD measurements are sensitive to the extension of optically coherent areas.

## 4. Conclusions

In this study, the effect of calcination temperature on the phase and microstructure evolution was investigated and the following summary can be made based on the results:

1. Proposed synthesis process effectively enhanced the reactivity of the substrates due to their nanometric size of boehmite. The nanocrystalline mayenite powders can be easily obtained using this method at temperatures of 470 °C a result of dehydration of the C3AH6 hydrogranate above 220 °C. Further portions of C12A17 are formed by the solid state reaction of the constituent oxides at higher temperatures;
2. It was found that calcination temperature is an important factor in the synthesis of nanocrystalline C12A7 powders, and the formation of aluminate phases depends on it. In the case of powders synthesized at lower temperatures, the presence of small amounts of Ca(OH)_2_ and CaO are observed which indicates only its partial decomposition under the heat treatment conditions. Reducing the proportion of these compounds after thermal reduction can occur through the washing of the precursors by 0.1 M NH_4_Cl/methanol solution mixture, which allows for their chemoselective dissolution [6];
3. Powders with a high BET surface area of around 71.18 m^2^/g containing C12A14H as the main phase showing 10.7 nm crystallite size can be synthesized followed by a relatively low temperature calcination of around 470 °C compared to the routine ceramic method involving solid state reaction, which requires over 1000 °C to form the same phase, but a very low BET surface area of below 10 m^2^/g [22]. Use of a novel synthetic route based on the use of PMMA as a soft templating agent [27] also does not give the possibility of synthesizing mayenite powders with such a large surface area as the hydrothermal method allows.

## Figures and Tables

**Table 1:** BET surface area and the average crystallite/particle size of CA powders.

Sample	S_BET_ (m^2^/g)	Crystallite/Particles Size [nm]	
		D_XRD_	D_TEM_
470 °C	71.18 ± 0.20	31.1	10.7
760 °C	50.26 ± 0.08	44.9	17.6
960 °C	10.34 ± 0.04	81.3	78.1
